# Identification and Growth Characterization of a Novel Strain of *Saccharomyces boulardii* Isolated From Soya Paste

**DOI:** 10.3389/fnut.2020.00027

**Published:** 2020-04-03

**Authors:** Md Nur Hossain, Sadia Afrin, Sanjida Humayun, Monzur Morshed Ahmed, Barun Kanti Saha

**Affiliations:** Institute of Food Science and Technology, Bangladesh Council of Scientific and Industrial Research, Dhaka, Bangladesh

**Keywords:** *Saccharomyces boulardii*, BIOLOG^TM^ identification, probiotic, antimicrobial activity, gastrointestinal disorders

## Abstract

The nonpathogenic yeast *Saccharomyces boulardii* (Sb) has beneficial effects on the human intestine, and thus has been prescribed as probiotics for the treatment of diarrhea and gastrointestinal diseases. This is the only commercialized yeast with the purpose of being used as human medicine. Currently, little is known about their multiple mechanisms of actions. The *S. boulardii* yeast strain is isolated and identified by using the BIOLOG^TM^ microarray identification system and morphologically. To understand its functional roles, the present study investigates the ability of this yeast to tolerate different concentrations of bile salt up to 2.5%, cell hydrophobicity, antioxidants, autoaggregation activity, and simulated gastrointestinal digestion. The effect of temperatures (up to 50°C), pH (up to 8.0), and salinity (at best 7%) was also monitored on the growth and survival of the yeast cell. The physicochemical analyses revealed that *S. boulardii* could survive in stomach conditions at pH 2.5, temperature 37°C, and 2% bile salt. Antibiotic susceptibility of *S. boulardii* was carried out using commercial antibiotic discs. The antimicrobial activity of the isolated *S. boulardii* against bacterial pathogens related to diarrhea diseases was *in-vitro* determined by the Well Diffusion method. The biosafety assay findings also claimed *S. boulardii* could be a potential probiotic. The experimental findings suggest that the isolated *S. boulardii* possesses excellent probiotic capacities as a biotherapeutic agent for antidiarrheal and gastrointestinal disorders.

## Introduction

*Saccharomyces boulardii* (Sb) is a nonpathogenic yeast initially isolated by French scientist Henri Boulard from the fruits lychee and mangosteen in 1923 (1). Earlier reports indicated that *S. boulardii* is a strain of *Saccharomyces cerevisiae*, sharing high level (~99%) genomic relatedness (2). Further taxonomic, metabolic, and genetic properties analysis revealed that the two strains have different genetic composition and enzyme profiles (1, 3). Moreover, *S. boulardii* has a unique and specific microsatellite allele characteristics that distinguishes it from other strains of *S. cerevisiae* (4). Characterization of *S. boulardii* as a separate species was additionally supported by the lack of galactose utilization and sporulation as compared to *S. cerevisiae* (5). Notably, *S. boulardii* is a thermotolerant yeast that grows optimally at 37°C (physiological temperature of the host), whereas *S. cerevisiae* strains grow and metabolize at 30°C (6, 7). Moreover, *S. boulardii* is an acid-tolerant yeast that secretes some distinct physiologically active factors. Some recent studies have demonstrated that *S. boulardii* appears to be more resistant than the *S. cerevisiae* strain when exposed to a simulated gastric environment (8, 9). *S. boulardii* is the only probiotic yeast whose effect has been evaluated in double-blind clinical studies. Importantly, *S. boulardii* has demonstrated clinical and experimental effectiveness in gastrointestinal diseases with a predominant inflammatory component (5, 10). For these unique characteristics, *S. boulardii* is widely used as a probiotic (8, 11, 12).

Probiotics are a particular group of live microorganisms that maintain or improve intestinal microbial balance, thus promoting the health benefits of consumers (13, 14). To exhibit benefits to human health, probiotics have to fulfill several criteria (14, 15). It must have excellent technological properties so that it can be manufactured and incorporated into food products without losing viability and functionality (16, 17). It has to survive through the upper gastrointestinal (GI) tract and arrive alive at its site of action, as well as being able to function in the gut environment (6, 18). Several recent studies indicated that the yeast *S. boulardii* could survive in a wide range of temperatures and pH levels (19, 20). Besides, *S. boulardii* has a strong affinity to various stresses like the presence of GI enzymes, bile salts, and organic acids (19, 21). *S. boulardii* benefits the GI tract in a variety of ways. It inactivates bacterial toxins, inhibits toxin binding to intestinal cell receptors, and reduces toxin-induced inflammation (22, 23). It inhibits the ability of potentially harmful microorganisms and invades intestinal cells (9). *S. boulardii* stimulates host immune systems and intestinal enzymes that enhance nutrient digestion and absorption (24). In support of these distinctive mechanisms, the live yeast *S. boulardii* is used extensively as a probiotic and is often marketed as a dietary supplement (10, 25). However, *S. boulardii* belongs to the group of simple eukaryotic cells; it thus differs from bacterial probiotics that are prokaryotes. In a study, it was reported that *S. boulardii* CNCM I-745 influences the gut-associated immune system (26). Furthermore, the previous reports indicated that this yeast decreased the incidence of antibiotic-associated diarrhea (9, 26) and diminished the risk of recurrence of *Clostridium difficile*-associated and Crohn's diseases (27). *S. boulardii* was also used for lactose intolerance, urinary tract infections (UTIs), vaginal yeast infections, high cholesterol levels, fever blisters, and teenage acne (25, 28, 29). Considering the beneficial aspects, the present study was undertaken to isolate and characterize the yeast *S. boulardii* and investigate its ability to tolerate different concentrations of bile salt, cell hydrophobicity, antioxidants, autoaggregation activity, and simulated gastrointestinal digestion. The probiotic activities were conducted in response to a different temperature, pH, and salinity (NaCl). Furthermore, the antagonistic spectrum of *S. boulardii* was analyzed using various bacterial pathogens related to diarrhea diseases. To the best of our knowledge, this is the first comprehensive report on a promising *S. boulardii* strain in Bangladesh. The experimental findings will give new insight for immunoblastic uses of *S. boulardii* as a therapeutic agent.

## Materials and Methods

### Sample Collection

About eight fresh soya paste samples from different sources that were available in local markets in Dhaka city, Bangladesh, were used for the isolation of *S. boulardii*. Collected samples were kept in sterilized bags and immediately brought to the laboratory and were preserved before and after analyses.

### Yeast Isolation and Screening

For the isolation of yeast, the pour plate technique was followed by using the Sabouraud dextrose agar (SDA) medium (Difco, Thermo Fisher Scientific). Inoculated plates were incubated at 28°C for 3–5 days (30). After incubation of the inoculated plates, developed colonies were observed. The isolates were first classified based on morphological characteristics and then tested for their ability to grow at higher temperatures (37°C). The selected isolates were purified by using the streaking method and preserved in the refrigerator at 4°C for further use.

### Microscopic Observation

To determine the ascospore formation and budding or splitting, lactophenol cotton blue reagent (Thermo Fisher Scientific) was used for staining as well as for wet mounting of yeast (31). A drop of lactophenol cotton blue reagent was placed on a clean and dry slide. By using a nichrome inoculating loop, the yeast culture is then carefully teased into a thin preparation. A coverslip is placed on the preparation and then left for about 3–5 min. The slides are then observed first under low power objectives for screening in low intensity and then observed under high power objectives.

### Identification of Yeast

#### Identification by Biochemical Characteristics

Several biochemical tests such as carbon source fermentation, nitrogen source utilization, acid production from fermented sugars, ester production, gelatin liquefaction, urea hydrolysis and H_2_S test were carried out as mentioned by van der Aa Kühle and Jespersen (1) and Jespersen (32).

#### Identification by BIOLOG^TM^ System

For the species-level identification, the BIOLOG^TM^ identification system was applied (BIOLOG^TM^, USA) based on the utilization of carbon sources and chemical sensitivity assays in yeast identification microplate (YT) test panels (11, 33). All the chemicals, reagents, and growth medium were supplied by (BIOLOG^TM^, USA). This system is based around a 96-well microtiter tray containing a range of dehydrated carbon sources for assimilation and oxidation tests such as assimilation of glucose, galactose, L-sorbose, sucrose, maltose, cellobiose, trehalose, lactose, melibiose, raffinose, melezitose, inulin, soluble amides, D-xylose, L- and D-arabinose, D-ribose, L-rhamnose, D-glucosamine, N-acetyl-D-glucosamine, methanol, ethanol, glycerol, erythritol, ribitol, galactitol (dulcitol), D-mannitol, D-sorbitol, μ-methyl-D-glucoside, salicin, D-gluconic acid, DL-lactic acid, sodium succinate, sodium citrate, inositol, hexadecane, μ-ketoglutaric acid, xylitol, L-arabinitol, propane 1.2 diol, butane 2.3 diol, lysine, ethylamine, potassium nitrate, cadaverine, creatine, and glucosamine. The other tests were: fermentation of glucose, sucrose, maltose, galactose, raffinose, lactose, trehalose, melezitose, cellobiose and inulin, starch formation, and so on. This system also maintains one positive and one negative control for comparison.

Before use, yeast isolates were cultured on Biolog universal yeast agar (BUY) medium and incubated at 30°C for 48 h. All microplates and inoculating fluid were pre-warmed at 30°C for 30 min. After 48 h incubation, the inoculum of yeast isolates was added to the inoculating fluid to obtain the desired turbidity, which is 44–51% T because the target cell density must be in the range of 44–51% T for YT microplate. Repeating Pipettor was filled by drawing up the cell suspension from the reservoir and all 96 wells were inoculated with 100 μl of the suspension. The microplate was then covered with its lid and incubated at 30°C for 24–48 h. After incubation, the microplate was placed into the Micro Station Reader for analysis to obtain ID result and the result was given through comparison with the database using the software program MicroLog 4.20.05 (BIOLOG™, USA) (33, 34). The scope of the 96 assay reactions, coupled with sophisticated interpretation software, delivers a high level of accuracy that is comparable to molecular methods.

### *In-vitro* Probiotic Attributes of *S. boulardii*

For the determination of probiotic properties, several major selection criteria were carried out, such as tolerance against a range of temperatures, bile salt, simulated gastrointestinal digestion, hydrophobicity of cell, autoaggregation and salinity (NaCl), as well as resistance to low pH (4). According to the suggestion of FAO/WHO, it is mandatory to perform a preliminary *in vitro* assessment before assessing the probiotic properties of microorganisms (14, 25). In the current study, some technological and probiotic properties of the selected strain were investigated.

### Thermal Stability Assay

To determine the thermal stability of the yeast strain, SDB (Sabouraud dextrose broth) medium (Difco, Thermo Fisher Scientific) was prepared and transferred to tubes in 5 ml. An inoculum of yeast strain was prepared in SDB medium by overnight incubation and 1% (v/v) fresh culture was added to each tube and incubated for 24, 48, and 72 h at 25, 30, 35, 37, 40, 45, and 50°C. After incubation, the survival rate of the isolate was measured by taking absorbance at 660 nm by a spectrophotometer (Thermo Multiskan EX) (19, 35).

### Bile Salt Tolerance

Growth rate of yeast culture was determined in SDB medium supplemented with different levels of bile salt concentrations (0.5, 1.0, 1.5, 2.0, and 2.5%). An inoculum of yeast strain was added to each tube containing bile salt after sterilization. The broth was then incubated at 30°C for 24, 48, and 72 h. The survival rate was measured by taking absorbance at 660 nm (7, 19).

### Resistance to pH

Acid tolerance of the yeast culture was studied by incubating the organisms in the SDB medium. Resistance to pH 3.0 is often used *in vitro* assays to determine the resistance to stomach pH. For this purpose, active culture was used to determine growth in different pH. 100 μl fresh culture of the isolate was inoculated into SDB medium with varying pH levels, ranging from 1.0 to 8.0. The low pH value was adjusted by using 1 M HCl (7, 28).

### Salinity (NaCl) Stability Assay

The yeast isolate was tested for tolerance against different NaCl concentrations. The growth rate of yeast culture in the SDB medium containing different levels (1–7%) of NaCl was determined (35).

### Resistance to Simulated Gastrointestinal Digestion

The solutions for simulated gastrointestinal digestion were prepared according to the method of Minekus et al. (36). During the gastrointestinal digestion, the survivability of the yeast strain was assessed using *in-vitro* digestion system. The *in vitro* gastrointestinal digestion model applied in this study consisted of a three-step procedure, which sequentially simulated the digestion in the mouth, stomach, and the small intestine as described by Minekus et al. (36) and Gut et al. (9), with some modification. All the chemicals and enzymes were purchased from Sigma Aldrich, USA. In this method, simulated Silivary Fluid (SSF) was prepared by dissolving 0.1126 g KCL, 0.0503 g KH_2_PO_4_, 0.1142 g NaHCO_3_, 0.0030 g MgCL_2_(H_2_O)_6_, and 0.0006 g (NH4)_2_CO_3_ in 70 ml Milli-Q water, adjusted pH to 7.0. Simulated Gastric Fluid (SGF) was prepared by dissolving 0.0514 g KCL, 0.0122 g KH_2_PO_4_, 0.210 g NaHCO_3_, 0.2761 g NaCl, 0.0024 g MgCL_2_(H_2_O)_6_, and 0.0048 g (NH4)_2_CO_3_ in 70 ml Milli-Q water, adjusted pH to 3.0. Simulated Intestinal Fluid (SIF) was prepared by dissolving 0.0507 g KCL, 0.0108 g KH_2_PO_4_, 0.714 g NaHCO_3_, 0.2246 g NaCl, and 0.0067 g MgCL_2_(H_2_O)_6_ in a 70 ml Milli-Q water, adjusted pH to 7.0. The samples were incubated in a shaking incubator at 37°C and the colonies were counted accordingly. α-amylase from human saliva, porcine pepsin, and porcine pancreatic lipase enzymes were used for mouth mastication, gastric, and intestinal digestion, respectively.

### Hydrophobicity

The cell surface hydrophobicity was evaluated with chloroform and n-hexadecane. Cells were suspended in 3 ml of a 50 mM potassium phosphate buffer at pH 7.0 (23, 37). The suspensions were centrifuged at 10,000 × g for 5 min at 4°C. Pellets were collected, washed twice, and then resuspended in the same buffer. Absorbance at 600 nm was measured and considered as A_0_. One milliliter of the suspension was mixed with 200 μl of chloroform and n-hexadecane by vortexing for 120 s. The two phases were allowed to separate for 1 h at room temperature. The lower aqueous layer was carefully transferred to clean tubes and absorbance was measured as A. Changes in the absorbance of probiotic bacterial suspension were recorded at 600 nm (Thermo Multiskan EX, Thermo Scientific, USA).

Surface hydrophobicity (*SHb*%) was determined using the following formula:

$$SHb\%=\frac{\text{A}0-\text{A}}{\text{A0}}\times 100$$

where A_0_ and A are the absorbances before and after extraction with chloroform and n-hexadecane, respectively. All *SHb* experiments were repeated 3 times for statistical analysis.

### Autoaggregation Ability

The yeast culture in the SDB medium was harvested by centrifugation (5,000×g, 5 min), washed, and resuspended with saline solution (0.85%) (19, 23). The absorbance was measured at 0, 2, and 24 h in a spectrophotometer (Thermo Multiskan EX, Thermo Scientific, USA) at 600 nm without shaking the cell suspension. The autoaggregation was calculated as follows:

$$\begin{array}{l} \text{Autoaggregation}\left(\text{\%}\right)=\left(1-\frac{At}{A0}\right)\;\times \;100 \end{array}$$

*A*_t_ is the absorbance at 600 nm and *A*_0_ is the initial absorbance at 600 nm.

### Assessment of Antibiotic Susceptibility

The selected strain was investigated for its antibiotic resistance profile as recommended by the Clinical and Laboratory Standards Institute (CLSI; Wayne, PA, USA). The yeast strain was grown overnight in the SDB medium at 30°C. Petri dishes containing 20 ml of Muller Hinton Agar (MHA) medium (Hi-Media, India) were allowed to solidify at room temperature and swabbed with inoculated SDB. In this study, 12 different types of antibiotic discs such as Ampicillin (10 μg), Amikacin (30 μg), Cephotaxime (30 μg), Ciprofloxacin (5 μg), Chloramphenicol (30 μg), Erythromycin (15 μg), Gentamicin (10 μg), Imipenem (10 μg), Kanamycin (30 μg), Metronidazole (5 μg), Rifampicin (30 μg), and Vancomycin (30 μg) were used. All the antibiotics were purchased from (Hi-Media, India). The agar plates were incubated at 30°C for 24 h. Diameter (in mm) of the inhibition zone was measured using an antibiotic zone scale. The results were expressed in terms of resistance, moderate susceptibility, or susceptibility by comparing with the interpretative zone diameters provided in the performance standards for antimicrobial disk susceptibility tests (9, 10).

### Antimicrobial Activity of *S. boulardii* Against Pathogenic Bacteria and Fungi

The selected strain was investigated for antimicrobial activity against a variety of pathogenic microorganisms. A modified agar well diffusion method was used to detect antimicrobial activities (10, 12, 19). These assays were performed in triplicate. The plates were poured with 20 ml MHA media. Ten (10) different pathogens belonging to both gram-positive and gram-negative groups such as *Salmonella typhi* ATCC 13311, *Bacillus megaterium* ATCC 13578, *Shigella flexneri* ATCC 12022, *Enterococcus faecalis* ATCC 29212, *Klebsiella Pneumoniae* ATCC 13883, *Enterobacter aerogenes* ATCC 13048, *Vibrio paraheamolyticus* ATCC 17802, *Escherichia coli* ATCC 11303*, Clostridium difficile* (lab culture) and *Helicobacter pylori* (lab culture), and three fungi named *Candida albicans* ATCC 28121, *Rhizopus oryzae* ATCC 22961, and *Apergillus parasiticus* (lab culture). All the ATCC cultures were collected from American type culture collection center. The pathogenic strains were grown in NB (Nutrient broth) medium (Hi-Media, India) for 24 h and spread on the surface in the MHA plate. Three wells (6 mm in diameter) of each plate were made by using a sterile borer and 10 μl of the supernatant of the *S. boulardii* was placed into three wells. The plates were pre-inoculated at room temperature for the diffusion and incubated overnight at the respective temperatures. The plates were examined for zones of inhibition. At the end of the incubation, the diameters of inhibition zone were measured. Accordingly, the isolates that gave an inhibition zone bigger than 1 mm were determined to have antimicrobial activity. All the test isolates of bacteria and fungi were taken from the culture collection pool of Industrial Microbiology Laboratory, IFST, BCSIR, Dhaka.

### Antioxidant Activity

The antioxidant capacity of *S. boulardii* and autolysates were assayed as described by Fakruddin et al. (10, 12), with some modifications. Fresh yeast culture was harvested by centrifugation (5,000×g, 10 min), washed, and resuspended in saline solution (0.85% NaCl). The yeast suspension (800 μL) was transferred into a new tube, 1 mL of DPPH (Sigma Aldrich, USA) solution (0.2 mM in methanol) was added, vortexed, and incubated at room temperature safe from light. After 30 min, the tube was centrifuged (3,000×g, 10 min) and 200 μL of the supernatant was transferred into 96-well plates to measure the absorbance at 517 nm. The reduction of DPPH was expressed as a percentage calculated according to the following equation:

$$\begin{array}{l} \text{Reduction of DPPH }\left(\text{\%}\right)=\left(1-\frac{A}{A0}\right)\;\times \;100 \end{array}$$

A is the absorbance of the sample at 600 nm and A_0_ is the blank absorbance at 600 nm.

Results were classified as low activity (20–30%), good activity (30–40%), very good activity (40–50%), and excellent activity (>50%) according to Gil-Rodríguez et al. (12) and Romero-Luna et al. (19).

### *In-vivo* Antidiarrheal Activities in Mice

Sixteen male swiss albino mice aged 5–6 weeks were divided into four treatment groups designated as C_1_, C_2_, T_1_, and T_2_ (four mice in each group). C_1_ is for control with normal feed, C_2_ is for control with induced diarrhea with normal feed, T_1_ is for normal feed with *S. boulardii*, and T_2_ is for induced diarrhea with normal feed and *S. boulardii*. Castor oil was induced orally (2 ml) and mice become infected with diarrhea by 7/8 h. A single dose ~7 log CFU/g *S. boulardii* was administered orally to the test group with normal feed. The control group mice were fed with normal feed. The test mice were monitored daily for 10 days to observe any changes in their activities, behavior, and general health. Before feeding in the morning, individual body weight was recorded and feces were collected daily to enumerate the total numbers of *S. boulardii* and Enterobacteria (6, 10). Enterobacteriaceae was taken into consideration as it is an indication of fecal contamination and could indicate the possible presence of enteric pathogens.

### Statistical Analysis

Data analyses were carried out with SPSS software (version 20.0; SPSS Inc., Chicago, USA). All experiments were carried out in triplicate. Data were presented as the mean ± standard deviation (SD) for the indicated number of independently performed experiments. *P* < 0.05 was considered statistically significant using a one-way analysis of variance (ANOVA).

## Results

### Yeast Isolation and Screening

Samples were diluted with sterile water and plated into the SDA medium and thenincubated at 30°C for 48 h of incubation. In this study, only one isolate was found to exhibit a strong affinity with high temperatures, and was successfully grown and survived at 37°C. As a result, this isolate initially presumes as *S. boulardii* and was chosen for further study.

### Morphological Determination by Microscopic Observation

**Figure 1:**
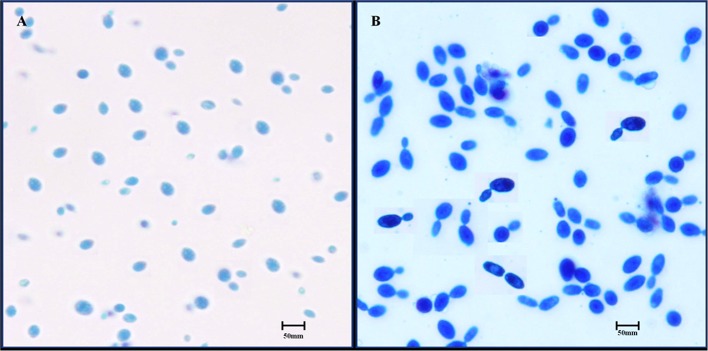
Microscopic observation of *S. boulardii* cells. **(A)** Oval and/or spherical shapes under low power objective. **(B)** Budding of *S. boulardii* under high power objective.

The selected isolate was observed by an optical microscope to determine their morphological characteristics. The isolated colony was found to have smooth surfaces with circular margins, a creamy white color, and was oval and/or spherical in shape (Figure 1A). When the strain staining with lactophenol cotton blue staining was observed under high power objective, it showed clear budding (Figure 1B).

### Identification and Characterization of *S. boulardii*

From the last two decades, a variety of methods have been demonstrated for yeast identification. In this study, yeast was initially identified based on physiological and morphological views and finally confirmed by biochemical characteristics as well as BIOLOG™ identification system.

### Identification by Biochemical Characteristics

Important biochemical tests were carried out for the provisional identification of the yeast strain. The results indicated that the selected strain can utilize the majority of carbon and nitrogen sources. The strain has given brown and black color colonies on the bismuth sulfite agar medium, indicating hydrogen sulfite production (Table 1).

**Table 1:** Biochemical characteristics of the *S. boulardii* strain.

**Tests**		**Result**
Nitrogen utilization	Nitrate	–
	Peptone	+
	Ammonium sulfate	+
Carbon utilization	Glucose	+
	Fructose	+
	Sucrose	+
	Lactose	–
	Starch	+
Acid production		+
Ester production		+
Urea hydrolysis		–
Gelatin Liquefaction Test		–
H_2_S Test		Brownish, Black

### Identification of Isolate Using the BIOLOGTM System

The strain was identified with the BIOLOG™ microarray identification system up to the species level. The BIOLOG^TM^ system aims to provide a rapid, convenient approach to yeast identification with a database of 267 species. The result indicates that the yeast consistently identified as *S. boulardii* (Supplementary Figure 1). For further confirmation, these isolates were examined for three replications. Table 2 shows the result of the BIOLOG^TM^ identification system.

**Table 2:** Microorganism identification with BIOLOG^TM^ system.

**Organism type**	**ID (identification)**	**PROB (probability)**	**SIM (similarity index)**	**DIST (distance)**
Yeast strain	*Saccharomyces boulardii*	0.863	0.581	5.000

### Probiotic Properties of *S. boulardii*

#### Thermal Stability Assay

**Figure 2:**
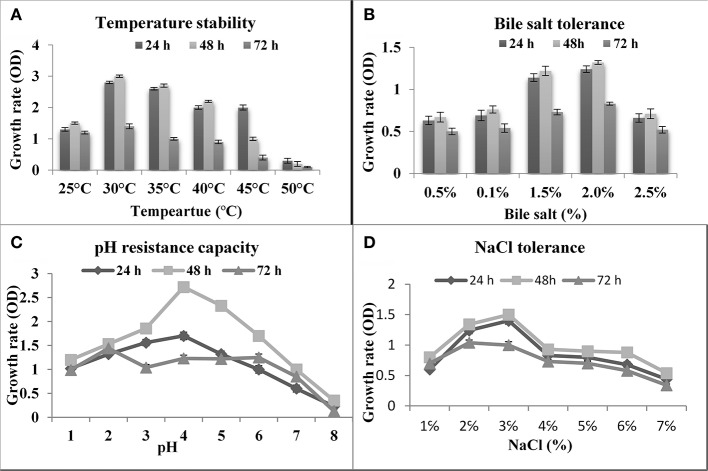
Effect of temperature, bile, pH and NaCl on the growth of S. boulardii. **(A)** Temperature stability. **(B)** Bile salt tolerance. **(C)** pH resistance capacity. **(D)** NaCl Tolerance.

Temperature is one of the most important factors for probiotic assortment. In rumen temperature is 42°C, therefore, if used, any probiotic organism in ruminants must tolerate 42°C. As shown in the present study, the isolated thermo-tolerant yeast *S. boulardii* is viable at 45°C (Figure 2A).

#### Bile Salt Tolerance

Bile is a dark green to a yellowish-brown fluid whose principal constituents includes bile acids, cholesterol phospholipids, and pigment biliverdin. It's synthesized in the pericentral hepatocytes of the liver and stored and concentrated in the gallbladder. As shown in Figure 2B, the isolated yeast *S. boulardii* had shown the best ability to tolerate bile salts and had reasonable growth rate even in 2% bile salt.

#### pH Resistance Assay

The first barrier in the gut is the acidic condition in the stomach. In this work, the abilities of the *S. boulardii* strain to endure low pH were investigated. The yeast strain successfully survived at pH 2.0 and up (Figure 2C). Besides, the pH of the small intestine is 8.5, so the isolated organism is tested on alkaline pH. The obtained result indicated that an isolated *S. boulardii* strain can survive up to pH 8.0.

### Effect of NaCl Tolerance

Tolerance to sodium chloride was determined by testing their ability to grow in the presence of different concentrations of NaCl. According to the result of the test, the isolate was growing up to 8.0% NaCl concentration, but there was a rapid decrease in growth after 3.0% NaCl concentration (Figure 2D).

### Simulated Gastrointestinal Tolerance

**Figure 3:**
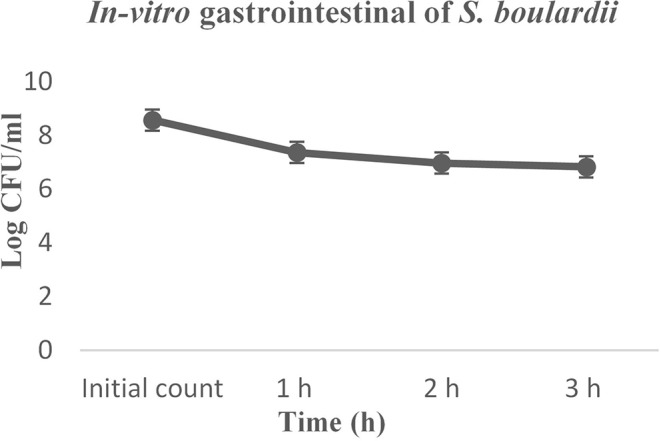
*In-vitro* gastrointestinal digestion effect on the growth of *S. boulardii*.

Before reaching the intestinal tract, probiotic yeast must survive transit through the stomach and exposure to gastric acid constituents, which is a primary defense mechanism against most ingested microorganisms. The simulated gastrointestinal digestion included mouth mastication, gastric digestion, and small intestine digestion at 37°C for 3 h. In the current study, the *S. boulardii* strain exhibited strong resistance to gastric juice (Figure 3). The initial count was 8.562 log CFU/g and, at the end of the digestion, almost 80% *S. boulardii* had survived.

### Cell Hydrophobicity and Autoaggregation Activity

The hydrophobic properties of *S. boulardii* showed a greater affinity to chloroform compared to n-hexadecane, which are 40.31 and 23.11%, respectively. The autoaggregation ability of *S. boulardii* increases over time. The rapid autoaggregation obtained showed percentages of 54.76% at 2 h and 97.35% at 24 h.

### Antibiotic Susceptibility Profile

The antibiotic susceptibility of *S. boulardii* was carried out by using diverse commercial antibiotics and the obtained results were compared with standard duffusion susceptibility test protocol (38). The findings indicated that the isolate was sensitive to some antibiotics and resistant to others (Table 3).

**Table 3:** Antibiotic susceptibility profile of *S. boulardii* isolate.

**Name of antibiotics**	**Potency**	**Zone of inhibition (mm)**	**Sensitivity**
Cefotaxime	30 μg	23.0	S
Ciprofloxacin	5 μg	21.0	S
Erythromycin	15 μg	24.0	S
Imipenem	10 μg	15.0	R
Kanamycin	30 μg	19.0	S
Chloramphenicol	30 μg	14.0	MS
Rifampicin	30 μg	19.0	MS
Vancomycin	30 μg	13.0	MS
Ampicillin	10 μg	8.0	R
Amikacin	30 μg	12.0	R
Gentamicin	10 μg	16.0	S
Metronidazole	5 μg	24.0	S

*MS, moderately sensitive; R, resistant; S, sensitive*.

### Antimicrobial Activities of *S. boulardii* Against Bacterial and Fungal Pathogens

To obtain the antimicrobial activity of *S. boulardii* against pathogenic bacteria, ten most common opportunistic and pathogenic bacteria (both gram-positive and gram-negative) related to diarrheal conditions and three fungi were used in this study as test pathogens. The average diameters of inhibition zones were given in Table 4. The mode of action (bacteriostatic or bactericidal) of *S. boulardii* isolate on test organisms was also determined according to the Ocampo et al. (39). Usually, bacteriostatic means that the agent prevents the growth of bacteria (i.e., it keeps them in a stationary phase of growth) and bactericidal means that kills bacteria. Although *S. boulardii* exhibits a wide range of zone of inhibition, it was bacteriostatic for most of the pathogen.

**Table 4:** Antimicrobial activity of *S. boulardii* against test pathogens, inhibition zone measured in mm (mean ± SD).

**Test organism**	**Zone of inhibition (mm)**	**Mode of action**
**GRAM-POSITIVE PATHOGENIC BACTERIA**		
*Enterococcus faecalis*	22.0 ± 0.30	Bs
*Bacillus megaterium*	30.0 ± 0.21	Bc
*Clostridium difficile*	23.0 ± 0.26	Bs
**GRAM-NEGATIVE PATHOGENIC BACTERIA**		
*Shigella flexinery*	21.0 ± 0.24	Bs
*Salmonella typhi*	26.0 ± 0.28	Bc
*Vibrio parahemolyticus*	22.5 ± 0.31	Bs
*Escherichia coli*	24.5 ± 0.28	Bs
*Klebsiella pneumonia*	20.5 ± 0.29	Bs
*Enterobacter aerogenes*	22.0 ± 0.32	Bs
*Helicobacter pylori*	24.0 ± 0.27	Bs
**FUNGI**		
*Candida albicans*	17.6 ± 0.13	Bs
*Rhizopus oryzae*	23.5 ± 0.27	Bs
*Apergillus parasiticus*	18.3 ± 0.19	Bs

*Bc, Bactericidal, Bs, Bacteriostatic*.

### Antioxidant Activity

The probiotic *S. boulardii* demonstrated a very good antioxidant activity with a reduction of DPPH percentage 46.48 ± 0.31%. The control ascorbic acid and *S. cerevisiae* showed an excellent antioxidant activity which was 66.23 ± 0.12% and 62.76 ± 0.05%, respectively.

### *In-vivo* Antidiarrheal Activities for Safety Evaluation

During the trial, all mice were observed carefully by their locomotor behavior and physical parameters: stool, urine, water consumption, and body temperature. The body weight between probiotic fed mice and control mice differed significantly. For induced diarrheal mice there were significant differences in stool conditions and body weight as well. At the end of the trial, the count of *S. boulardii* and Enterobacteria clearly indicated the antidiarrheal effect of *S. boulardii* (Table 5).

**Table 5:** *S. boulardii* and Enterobacteria counts along with feces conditions in tested the mice feces.

**Group**	**Parameters**	**Days (Log CFU/g)**			
		**0**	**3**	**7**	**10**
C_1_	*S. boulardii*	0	0	0	0
	Enterobacteria	4.65 ± 0.10	5.46 ± 0.29	6.73 ± 0.19	7.10 ± 0.16
	Feces conditions	Normal	Normal	Normal	Normal
C_2_	*S. boulardii*	0	0	0	0
	Enterobacteria	4.25 ± 0.38	4.72 ± 0.07	5.95 ± 0.21	6.81 ± 0.30
	Feces conditions	Normal	Normal	Normal	Normal
T_1_	*S. boulardii*	6.92 ± 0.19	7.64 ± 0.13	8.23 ± 0.17	9.87 ± 0.39
	Enterobacteria	4.64 ± 0.11	5.84 ± 0.22	5.20 ± 0.06	3.92 ± 0.14
	Feces conditions	Watery	Watery	Looser	Normal
T_2_	*S. boulardii*	7.10 ± 0.26	7.52 ± 0.21	8.20 ± 0.34	9.30 ± 0.21
	Enterobacteria	4.70 ± 0.06	5.12 ± 0.23	4.84 ± 0.14	3.70 ± 0.09
	Feces conditions	Watery	Normal	Normal	Normal

## Discussion

In the present study, only one isolate was found to exhibit a strong affinity with high temperatures, and was able to successfully grow and survive at 37°C (1, 32) since the temperature can differentiate *S. boulardii* from *S. cerevisiae* (2, 4). As a result, this isolate initially presumes as *S. boulardii* and was chosen for further study. The microscopic studies of the isolate showed that the isolated colony had smooth surfaces with circular margins and was a creamy white color and hadoval and/or spherical shapes (31). Furthermore, *S. boulardii* was subjected to lactophenol cotton blue staining and observed budding. The yeast strain was initially identified based on physiological and morphological views and finally confirmed by biochemical characteristics (3, 40, 41), as well as BIOLOG™ identification system.

The result of the biochemical tests indicated that the selected strain can utilize the majority of carbon and nitrogen sources (Table 1). The present study also complies with the same phenomenon as previous studies (20, 42). The strain was identified with BIOLOG™ microarray identification system up to the species level (Table 2). For further confirmation, these isolates were examined for three replications (11, 33, 34).

In the current study, some technological and *in vitro* probiotic properties of the selected strain were investigated according to the suggestion of FAO/WHO (14, 25). As shown in Figure 2A, the isolated yeast strains have a strong ability to tolerate high temperatures and *S. boulardii* is viable at 45°C, which agrees with the previous reports (35). Bile resistance is an important criterion in the selection of culture as a dietary adjunct because it could allow the growth of the ingested probiotic microorganism in the intestinal tract (6, 7). As shown in Figure 2B, the isolated yeast *S. boulardii* had shown the best ability to tolerate bile salts and had reasonable growth rate even in 2% bile salt. The previous study reported that the growth of probiotic yeast strains can survive up to 2% bile salts (4, 7). The first barrier in the GIT is the acidic condition in the stomach and the *S. boulardii* strain successfully survived at pH 2.0 and up to pH 8.0 (Figure 2C). Several previous reports strongly support the present results (4, 12). According to the result of the test (Figure 2D), the isolate was growing up to 8.0% NaCl concentration, but there was a rapid decrease in growth after 3.0% NaCl concentration (25).

Simulated gastrointestinal juice tolerance is considered one of the major criteria for probiotics, which is a primary barrier against most ingested microorganisms (19, 43). The current results thus revealed that the *S. boulardii* strain exhibited strong resistance to gastric juice (Figure 3) in pH 3.0 and almost 80% of cells successfully survived, with some previous study data strongly supporting the results (5, 9, 18). The cell hydrophobic and autoaggregation properties in the intestinal epithelial cells are considered as a probiotic criterion. The *S. boulardii* showed a good hydrophobic affinity and autoaggregation ability increases over time, which matches the previous study result (19, 43).

The obtained result indicated that the isolate was sensitive to or moderately sensitive to diverse commercial antibiotics, which is one of the criteria to be probiotics (10, 31, 44). The previous studies indicated that the strains *S. boulardii* possess good antagonistic activity against a broad spectrum of microorganisms (31, 44). The outer membrane of *S. boulardii* is rich in mannose, allowing pathogens that contain type-1 pili (mannose-binding fibers) in their structure to bind this mannose rich membrane, and this binding action prevents *E. coli* and other harmful bacteria from adhering to intestinal cells (20). In this connection, many investigators discussed the unique vital role of *S. boulardii* against pathogen infection. The result (Table 4) revealed that *S. boulardii* exhibited various degrees of inhibitory activities against the gram-positive and gram-negative pathogenic bacteria, indicating a broad spectrum of inhibition patterns of this organism (18, 29), as well as pathogenic fungus. Maximum inhibitory activity was observed against *Bacillus megaterium*, followed by *Salmonella* sp and *E. coli*. On the other hand, *S. boulardii* showed the least susceptibility to *Enterobacter aerogenes and Vibrio parahemolyticus*. The S. boulardii is moderately susceptible to pathogenic fungus. More than 12 mm zones of inhibition against other organisms were considered as susceptibile to this isolate (26, 45). *S. boulardii* exhibits a wide range of zone of inhibition and it was bacteriostatic for most of the pathogen (4, 9, 25). The above result indicates that the isolate might produce some substances which would have an inhibitory effect for a wide range of bacteria. Besides, the probiotic *S. boulardii* demonstrated a very good antioxidant activity with a reduction of DPPH reduction percentage (10).

**Figure 4:**
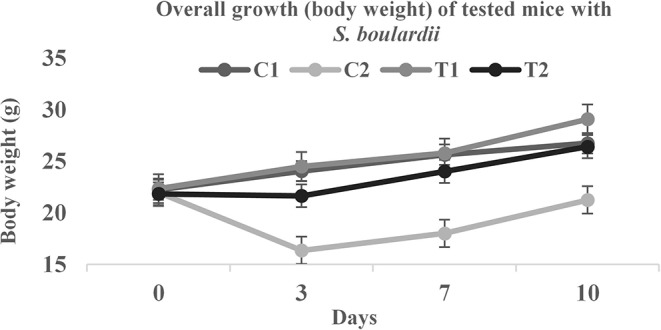
Body weight comparison of tested mice.

Biosafety assessment of potential probiotics is an important criterion for therapeutic applications. To assess the acute toxicity and normal growth behavior, the testing mice were fed with added *S. boulardii*. There were significant differences found in overall growth and diarrheal status after the feeding period (Figure 4 and Table 5). In comparison to control, the average body weight increased by almost 9% with normal activities. The feces condition became normal within 3 days ingestion of *S. boulardii* and lost weight was regained very quickly. A significant number of *S. boulardii* was counted in the feces after 10 days of the trail in the treated group, which dominated over the growth of enterobacteria in the control group. The biosafety assessment results were supported by some other previous findings (10, 28, 29, 44).

## Conclusion

Results of the present investigation support that *S. boulardii* strains isolated from soy paste samples are free from potential virulence traits and sensitive to clinically relevant antibiotics. This *S. boulardii* isolate was found to be safe with excellent probiotic attributes and positive health effects, as it showed a relatively high tolerance to gastrointestinal stresses and exhibited significant antimicrobial activity. Hence, *S. boulardii* possess interesting probiotic properties that make them potentially good candidates for probiotics. However, further *in-vivo* clinical studies are necessary in order to evaluate their role in strengthening the immune response and lowering the diarrhea level.

## Data Availability Statement

The datasets generated for this study are available on request to the corresponding author.

## Ethics Statement

Experimental protocols for the animal trial was approved by the BCSIR institutional ethical review committee and followed while performing the research with mice.

## Author Contributions

MH planned the study. MH, SH, and SA performed all the experiments. MA and BS provided necessary advice and guidelines in conducting the work. MH wrote the first draft of the manuscript. All authors read and approved the final manuscript.

### Conflict of Interest

The authors declare that the research was conducted in the absence of any commercial or financial relationships that could be construed as a potential conflict of interest.

## References

[1] van der Aa Kühle A Jespersen L. The taxonomic position of *Saccharomyces boulardii* as evaluated by sequence analysis of the D1/D2 domain of 26S rDNA, the ITS1-5.8 S rDNA-ITS2 region and the mitochondrial cytochrome-c oxidase II gene. Syst Appl Microbiol. (2003) 26:564–71 10.1078/072320203770865873 14666985

[2] Fietto JL Araújo RS Valadão FN Fietto LG Brandão RL Neves MJ. Molecular and physiological comparisons between *Saccharomyces cerevisiae* and *Saccharomyces boulardii*. Can J Microbiol. (2004) 50:615–21. 10.1139/w04-050 15467787

[3] Edwards-Ingram L Gitsham P Burton N Warhurst G Clarke I Hoyle D. Genotypic and physiological characterization of *Saccharomyces boulardii*, the probiotic strain of *Saccharomyces cerevisiae*. Appl Environ Microbiol. (2007) 73:2458–67. 10.1128/AEM.02201-06 17293506 PMC1855594

[4] Czerucka D Piche T Rampal P. Review article: yeast as probiotics-*Saccharomyces boulardii*. Aliment Pharmacol Ther. (2007) 26:767–78. 10.1111/j.1365-2036.2007.03442.x 17767461

[5] Hudson LE McDermott CD Stewart TP Hudson WH Rios D Fasken MB. Characterization of the probiotic yeast *Saccharomyces boulardii* in the healthy mucosal immune system. PLoS ONE. (2016) 11:e0153351. 10.1371/journal.pone.0153351 27064405 PMC4827847

[6] Rajkowska K Kunicka-Styczynska A Probiotic activity of *Saccharomyces cerevisiae* var. boulardii against human pathogens. Food Technol Biotechnol. (2012) 50:230−6.

[7] Rajkowska K Kunicka-Styczynska A. Probiotic properties of yeasts isolated from chicken feces and kefirs. Pol J Microbiol. (2010) 59:257–63 10.33073/pjm-2010-039 21466043

[8] Suvarna S Dsouza J Ragavan ML Das N. Potential probiotic characterization and effect of encapsulation of probiotic yeast strains on survival in simulated gastrointestinal tract condition. Food Sci Biotechnol. (2018) 27:745–53. 10.1007/s10068-018-0310-8 30263800 PMC6049689

[9] Gut AM Vasiljevic T Yeager T Donkor O. Salmonella infection-prevention and treatment by antibiotics and probiotic yeasts: a review. Microbiology. (2018) 164:1327–44. 10.1099/mic.0.000709 30136920

[10] Fakruddin M Hossain MN Ahmed MM. Antimicrobial and antioxidant activities of *Saccharomyces cerevisiae* IFST062013, a potential probiotic. BMC Complement Altern Med. (2017) 17:64. 10.1186/s12906-017-1591-9 28109187 PMC5251302

[11] Banik A Mondal J Rakshit S Ghosh K Sha SP Halder SK Amelioration of cold-induced gastric injury by a yeast probiotic isolated from traditional fermented foods. J Funct Foods. (2019) 59:164–73. 10.1016/j.jff.2019.05.039

[12] Gil-Rodríguez AM Carrascosa AV Requena T Yeasts in foods and beverages: *in vitro* characterization of probiotic traits. LWT - Food Sci Technol. (2015) 64:1156–62. 10.1016/j.lwt.2015.07.042

[13] Sanders ME Merenstein D Merrifield CA Hutkins R Probiotics for human use. Nutr Bull. (2018) 43:212–25. 10.1111/nbu.12334

[14] FAO/WHO Guidelines for the Evaluation of Probiotics in Food. Report of a Joint FAO/WHO Working Group on Drafting Guidelines for the Evaluation of Probiotics in Food. London, ON (2002).

[15] Lin DC. Probiotics as functional foods. Nutr Clin Pract. (2003) 18:497–506. 10.1177/0115426503018006497 16215085

[16] Martín R Olivares M Marín ML Fernández L Xaus J Rodríguez JM. Probiotic potential of 3 lactobacilli strains isolated from breast milk. J Hum Lact. (2005) 21:8–17. 10.1177/0890334404272393 15681631

[17] Lahtinen SJ Gueimonde M Ouwehand AC Reinikainen JP Salminen SJ. Probiotic bacteria may become dormant during storage. Appl Environ Microbiol. (2005) 71:1662–3. 10.1128/AEM.71.3.1662-1663.2005 15746375 PMC1065127

[18] Romanin D Serradell M Maciel DG Lausada N Garrote GL Rumbo M. Down-regulation of intestinal epithelial innate response by probiotic yeasts isolated from kefir. Int J Food Microbiol. (2010) 140:102–8. 10.1016/j.ijfoodmicro.2010.04.014 20471126

[19] Romero-Luna HE Hernández-Sánchez H Ribas-Aparicio RM Cauich-Sánchez PI Dávila-Ortiz G. Evaluation of the probiotic potential of *Saccharomyces cerevisiae* Strain (C41) isolated from Tibicos by *in vitro* studies. Probiot Antimicrob Prot. (2019) 11:794–800. 10.1007/s12602-018-9471-2 30238220

[20] Perricone M Bevilacqua A Corbo MR Sinigaglia M. Technological characterization and probiotic traits of yeasts isolated from Altamura sourdough to select promising microorganisms as functional starter cultures for cereal-based products. Food Microbiol. (2014) 38:26–35. 10.1016/j.fm.2013.08.006 24290622

[21] Offei B Vandecruys P De Graeve S Foulquié-Moreno MR Thevelein JM. Unique genetic basis of the distinct antibiotic potency of high acetic acid production in the probiotic yeast *Saccharomyces cerevisiae* var. boulardii. Genome Res. (2019) 29:1478–94. 10.1101/gr.243147.118 31467028 PMC6724677

[22] Anoop V Rotaru S Shwed PS Tayabali AF Arvanitakis G. Review of current methods for characterizing virulence and pathogenicity potential of industrial *Saccharomyces cerevisiae* strains towards humans. FEMS Yeast Res. (2015) 15:57. 10.1093/femsyr/fov057 26195617

[23] Tiago FdCP Martins FdS Souza E Pimenta PFP Araújo HRC Castro IdM. Adhesion to the yeast cell surface as a mechanism for trapping pathogenic bacteria by Saccharomyces probiotics. J Med Microbiol. (2012) 61:1194–207. 10.1099/jmm.0.042283-0 22580913

[24] Ansari JM Colasacco C Emmanouil E Kohlhepp S Harriott O. Strain-level diversity of commercial probiotic isolates of Bacillus, Lactobacillus, and Saccharomyces species illustrated by molecular identification and phenotypic profiling. PLoS ONE. (2019) 14:e0213841. 10.1371/journal.pone.0213841 30901338 PMC6430388

[25] Palma ML Zamith-Miranda D Martins FS Bozza FA Nimrichter L Montero-Lomeli M. Probiotic *Saccharomyces cerevisiae* strains as biotherapeutic tools: is there room for improvement? Appl Microbiol Biotechnol. (2015) 99:6563–70. 10.1007/s00253-015-6776-x 26142388

[26] Stier H Bischoff SC. Influence of *Saccharomyces boulardii* CNCM I-745on the gut-associated immune system. Clin Exp Gastroenterol. (2016) 9:269. 10.2147/CEG.S111003 27695355 PMC5027949

[27] Hunt M Mather AE Sánchez-Busó L Page AJ Parkhill J Keane JA. ARIBA: rapid antimicrobial resistance genotyping directly from sequencing reads. Microb Genomics. (2017) 3:e000131. 10.1099/mgen.0.000131 29177089 PMC5695208

[28] Zanello G Meurens F Berri M Salmon HY. *Saccharomyces boulardii* effects on gastrointestinal diseases. Curr Issues Mol Biol. (2009) 11:47 18780946

[29] van der Aa Kühle A Skovgaard K Jespersen L. *In vitro* screening of probiotic properties of *Saccharomyces cerevisiae* var. boulardii and food-borne *Saccharomyces cerevisiae* strains. Inter J Food Microbiol. (2005) 101:29–39. 10.1016/j.ijfoodmicro.2004.10.039 15878404

[30] Fakruddin M Islam MA Quayum MA Ahmed MM Chowdhury N Characterization of stress tolerant high potential ethanol producing yeast from agro-industrial waste. Am J Biosci. (2013) 1:24–34. 10.11648/j.ajbio.20130102.11

[31] Das B Neha Nidhi R Roy P Muduli A Swain P Mishra S Antagonistic activity of cellular components of Bacillus subtilis AN11 against bacterial pathogens. Inter J Curr Microbiol Appl Sci. (2014) 3:795–809.

[32] Jespersen L. Occurrence and taxonomic characteristics of strains of *Saccharomyces cerevisiae* predominant in African indigenous fermented foods and beverages. FEMS Yeast Res. (2003) 3:191–200. 10.1016/S1567-1356(02)00185-X 12702452

[33] Kostas ET Cooper M Shepherd BJ Robinson JP Identification of bio-oil compound utilizing yeasts through phenotypic microarray screening. Waste Biomass Valorization. (2019) 1–13. 10.1007/s12649-019-00636-7

[34] Kostas ET White DA Du C Cook DJ. Selection of yeast strains for bioethanol production from UK seaweeds. J Appl Phycol. (2016) 28:1427–41. 10.1007/s10811-015-0633-2 27057090 PMC4789230

[35] Phong HX Klanrit P Dung NTP Yamada M Thanonkeo P Isolation and characterization of thermotolerant yeasts for the production of second-generation bioethanol. Ann Microbiol. (2019) 69:765–76. 10.1007/s13213-019-01468-5

[36] Minekus M Alminger M Alvito P Ballance S Bohn T Bourlieu C. A standardized static *in vitro* digestion method suitable for food - an international consensus. Food Funct. (2014) 5:1113–24. 10.1039/c3fo60702j 24803111

[37] Dianawati D Shah NP. Survival, acid and bile tolerance, and surface hydrophobicity of microencapsulated *B. animalis* ssp. lactis Bb12 during storage at room temperature. J Food Sci. (2011) 76:592–9. 10.1111/j.1750-3841.2011.02422.x 22416710

[38] Hudzicki J Kirby-Bauer Disk Diffusion Susceptibility Test Protocol (2009). American Society for Microbiology©2016 p. 1–23.

[39] Ocampo PS Lázár V Papp B Arnoldini M Zur Wiesch PA Busa-Fekete R. Antagonism between bacteriostatic and bactericidal antibiotics is prevalent. Antimicrob Agents Chemother. (2014) 58:4573–82. 10.1128/AAC.02463-14 24867991 PMC4135978

[40] Tiago FC Martins FS Rosa CA Nardi RM Cara DC Nicoli JR Physiological characterizationof non-Saccharomyces yeasts from agro-industrial and environmental origins with possible probiotic function. World J Microbiol Biotechnol. (2009) 25:657–66. 10.1007/s11274-008-9934-9

[41] Pennacchia C Blaiotta G Pepe O Villani F. Isolation of *Saccharomyces cerevisiae* strains from different food matrices and their preliminary selection for a potential use as probiotics. J Appl Microbiol. (2008) 105:1919–28. 10.1111/j.1365-2672.2008.03968.x 19120638

[42] Qvirist LA De Filippo C Strati F Stefanini I Sordo M Andlid T. Isolation, identification and characterization of yeasts from fermented goat milk of the Yaghnob Valley in Tajikistan. Front Microbiol. (2016) 7:1690. 10.3389/fmicb.2016.01690 27857705 PMC5093317

[43] Gut AM Vasiljevic T Yeager T Donkor ON Characterization of yeasts isolated from traditional kefir grains for potential probiotic properties. J Funct Foods. (2019) 58:56–66. 10.1016/j.jff.2019.04.046

[44] Rima H Steve L Ismail F. Antimicrobial and probiotic properties of yeasts: from fundamental to novel applications. Front Microbiol. (2012) 3:421. 10.3389/fmicb.2012.00421 23267352 PMC3525881

[45] Zavala L Golowczyc M Van Hoorde K Medrano M Huys G Vandamme P. Selected Lactobacillus strains isolated from sugary and milk kefir reduce Salmonella infection of epithelial cells *in vitro*. Beneficial Microbes. (2016) 7:585–95. 10.3920/BM2015.0196 27291404

