# Supplementary figures and images for: Identification and Growth Characterization of a Novel Strain of Saccharomyces boulardii Isolated From Soya Paste
Source: Front Nutr. 2020 Apr 3;7:27. doi: 10.3389/fnut.2020.00027 (PMC7145964; doi:10.3389/fnut.2020.00027)

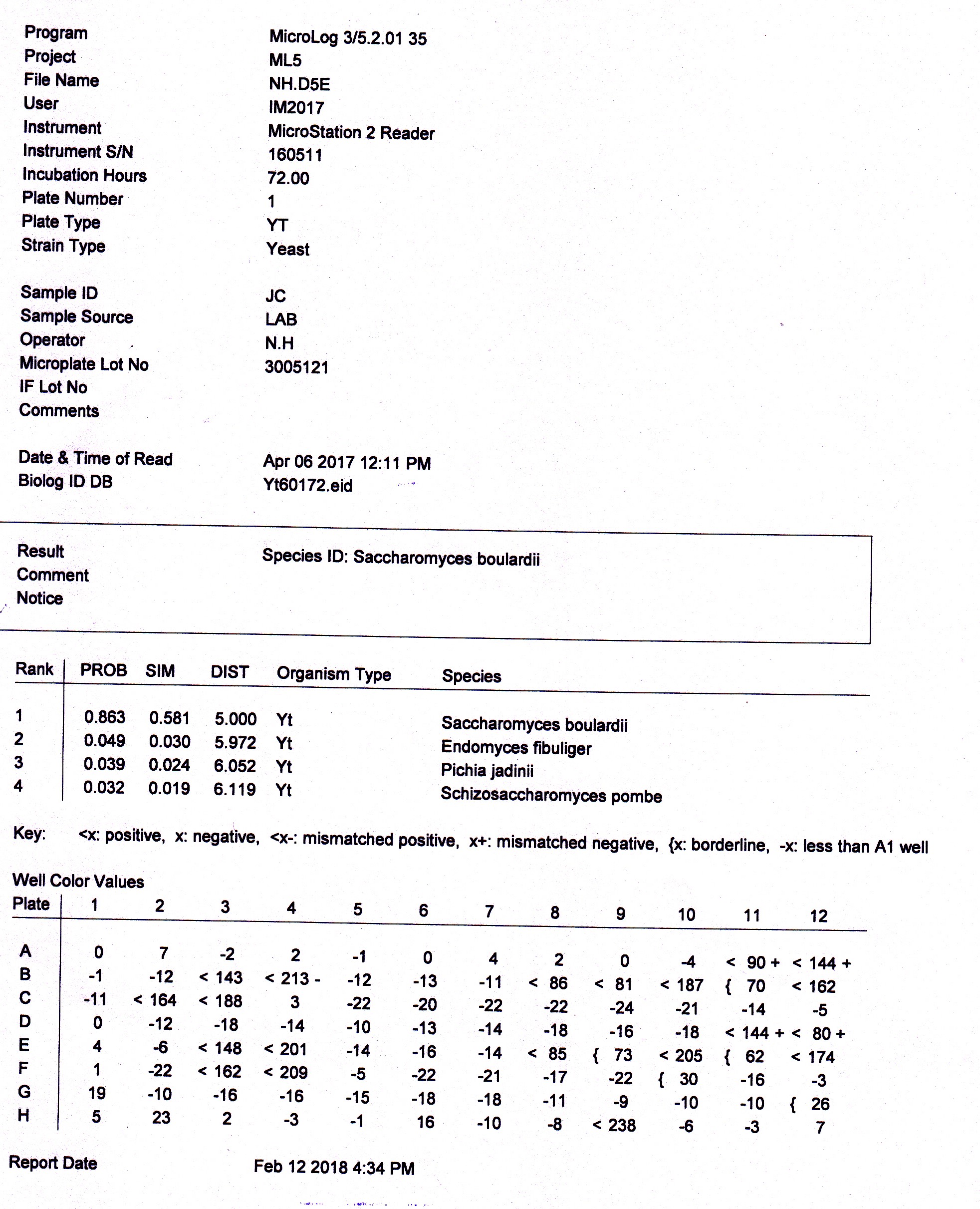

Supplement: Supplementary file 1 [file Image_1.jpg]
